# Transient Ischemic Attack in a Patient With Poland Syndrome With Dextrocardia

**DOI:** 10.7759/cureus.38185

**Published:** 2023-04-27

**Authors:** Iraj Afzal, Samin Rahman, Faiz Syed, Ofek Hai, Roman Zeltser, Amgad N Makaryus

**Affiliations:** 1 Internal Medicine, Nassau University Medical Center, East Meadow, USA; 2 Internal Medicine, New York Institute of Technology College of Osteopathic Medicine, Old Westbury, USA; 3 Cardiology, Nassau University Medical Center, East Meadow, USA; 4 Cardiology, Zucker School of Medicine at Hofstra/Northwell, Hempstead, USA; 5 Cardiology, Northwell Health, Manhasset, USA

**Keywords:** case report, dextrocardia, transient ischemic attack, poland syndrome, chatgpt

## Abstract

This report discusses the case of a patient with a past medical history of Poland syndrome and dextrocardia who was admitted for a transient ischemic attack. Poland syndrome is a rare genetic condition characterized by underdevelopment of chest wall musculature that presents with a variety of associations that may or may not be present in each case. This case report intends to discuss a unique presentation of Poland syndrome with dextrocardia, one of the rare conditions associated with Poland syndrome, as well as the treatment of Poland syndrome as a whole and possible associated complications.

## Introduction

Poland syndrome is a congenital condition characterized by varying thoracic abnormalities such as the absence of breast or nipple tissue, partial or complete absence of pectoral muscles, absence of costal cartilage in ribs 2-5, and lung hernia that is associated with chest wall defects [1]. It is a relatively rare condition affecting up to 1:100,000 births [2]. In some instances, it has been associated with dextrocardia, as seen in the patient presented in this case [3]. Poland syndrome is a congenital condition characterized by underdevelopment or absence of the pectoralis muscle on one side of the body, often associated with ipsilateral hand and arm malformations [4]. The most common presentation of Poland syndrome is an underdeveloped chest muscle on one side, with the affected side appearing smaller and less developed than the normal side. Other common features include webbing of the fingers or hand malformations on the affected side, and a difference in the size or shape of the breast on the affected side [5]. In rare cases, there may be additional malformations of the ribs, shoulder blade, or other muscles in the affected limb. The spectrum of symptoms can vary widely between individuals. Not all individuals with Poland syndrome will present with each of the symptoms. The patient presented in this case had degenerative changes of the spine and shoulder as well as dextrocardia. This case serves to better understand the patient's presenting symptoms as they tie in with Poland syndrome.

## Case presentation

A 51-year-old male with a past medical history of Poland syndrome, adrenal carcinoma, asthma, dextrocardia, and dyslipidemia presented to the hospital with complaints of dizziness, blurry vision, and numbness of the left lower and upper extremities with spontaneous resolution within 30 seconds. In addition, the patient also endorsed a throbbing headache on the right side without an associated visual aura.

Prior to experiencing the symptoms, the patient was playing a game of pickleball. He denied any cardiac history aside from dextrocardia. Furthermore, the patient denied fever, chest pain, shortness of breath, abdominal pain, blood in the urine, blood in the stool, syncope, incontinence, visual and auditory changes, and unintentional weight loss.

On initial evaluation in the Emergency Department, the patient was afebrile with a temperature of 97.9℉, heart rate of 84 beats per minute, respiratory rate of 18 breaths per minute, oxygen saturation of 100% on room air, and a blood pressure of 120/81.

**Figure 1:**
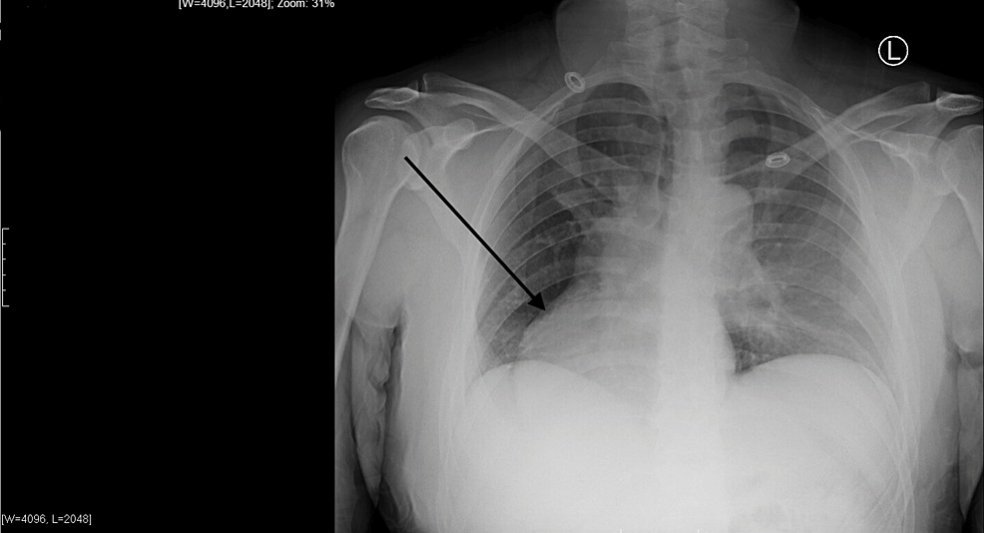
Chest X-ray demonstrating dextrocardia depicted by the arrow, degenerative changes of the bilateral shoulders, and chest wall asymmetry.

**Figure 2:**
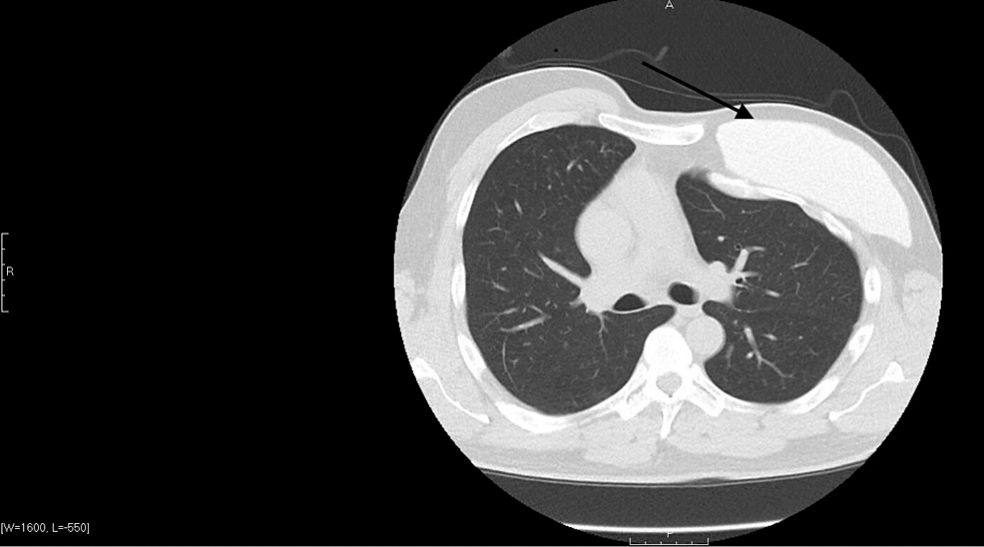
Chest CT without contrast demonstrating abnormal left chest wall musculature (depicted by the arrow), with a cosmetic left breast implant.

The patient’s initial chest X-ray and CT scan were negative for any acute pathological processes. Degenerative changes of the spine, bilateral shoulders, and left chest wall were noted on chest X-ray, as seen in Figure 1. Chest CT (Figure 2) further demonstrated the deformities of the left chest wall musculature with a left breast implant in line with the previous diagnosis of Poland syndrome.

The initial EKG showed sinus rhythm with a prolonged PR interval. The patient was admitted to the telemetry unit for cardiac monitoring and further workup of left-sided hemiparesis and paresthesias. The patient was further worked up with CT angiography of the head and neck, and an MRI of the brain, both of which did not show acute intracranial pathology, vessel occlusion, or infarction. A Doppler ultrasound of the bilateral carotid arteries did not show any evidence of stenosis. The patient was continued on cardiac monitoring and periodic neurological assessments, and daily EKGs, labs, and vitals were performed. The patient was continued on his home medications of hydrocortisone 10 mg every 12 hours and budesonide/formoterol 80-4.5 inhaler for adrenal insufficiency and asthma, respectively. He was started on aspirin 81 mg daily and rosuvastatin 20 mg daily, and following a Neurology consult, was also started on Plavix 75 mg daily for three weeks and advised to be discharged with a Holter monitor. The patient was medically stable and optimized for discharge with instructions to follow up with cardiology and pulmonology clinics within a week.

## Discussion

Poland syndrome is a rare congenital condition that affects around 1 in 30,000 to 1 in 100,000 newborns. The condition affects males and females equally, and it is not known to be inherited or caused by any specific environmental factors. Poland syndrome is typically a sporadic disorder, meaning that it is not inherited or passed down from parents to their children. However, in some cases, it has been reported to occur in more than one member of a family, suggesting a possible genetic component. It is also worth noting that Poland syndrome is often undiagnosed or misdiagnosed; hence, the true prevalence may be higher than what is reported in the literature. It is most commonly misdiagnosed as Duane-radial ray syndrome, which is characterized by eye anomalies and deficiencies of the upper limb [6]. Additionally, chest deformities may arise sporadically in conditions with extremity anatomical deformities such as Fanconi anemia, a hereditary disorder in which thumb and forearm malformation may be a presenting symptom. Fanconi anemia can be diagnosed through a complete blood count demonstrating pancytopenia with low reticulocyte count as well as a bone marrow tap demonstrating hypocellularity [7].

Dextrocardia is a congenital condition in which the heart is located on the right side of the chest instead of the left. In some cases, it may be associated with Poland syndrome, a congenital condition characterized by underdevelopment or absence of the chest muscle (pectoralis muscle) on one side of the body. It is worth noting that dextrocardia is a rare condition and is not commonly associated with Poland syndrome [8]. The combination of these two conditions is extremely rare and occurs in less than 1% of all cases of Poland syndrome. In individuals with dextrocardia and Poland syndrome, the right chest muscle is typically underdeveloped or absent, and the heart is located on the right side of the chest. This can lead to difficulty breathing due to a lack of accessory muscle assistance with respiration and may require surgical intervention to correct the position of the heart and improve breathing.

**Figure 3:**
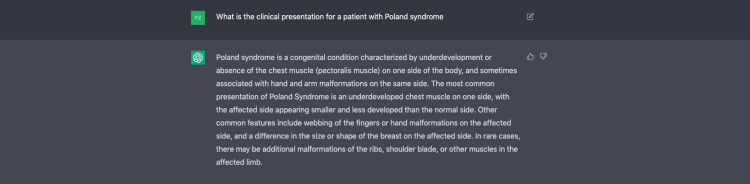
Utilizing ChatGPT for the clinical presentation of Poland syndrome.

**Figure 4:**
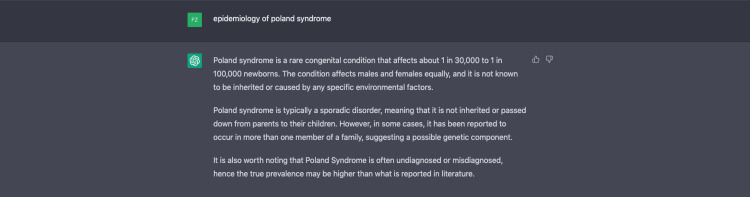
Utilizing ChatGPT for the epidemiology of Poland syndrome.

**Figure 5:**
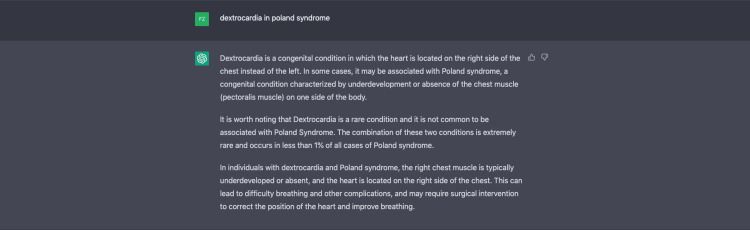
Utilizing ChatGPT to discuss dextrocardia in the setting of Poland syndrome.

The clinical presentation and epidemiology of Poland syndrome is written through ChatGPT. The authors’ experience using ChatGPT to write this section is demonstrated in Figures 3-5.

Although guidelines are not in place for the definitive treatment of Poland syndrome, surgical management can correct some of the major abnormalities associated with the condition. These procedures are geared toward cosmetic management, relief of visceral compression, and assistance in functional breathing. Thoracic surgery is usually related to rib cage abnormalities, and surgeries such as the Nuss procedure can lift up the sternum to relieve visceral compression [9].

This case is of particular interest because the patient presented with left-sided transient stroke-like symptoms. Previous literature has discussed a possible correlation between dextrocardia and left-sided Poland syndrome [8,10]. The left-sided structural abnormalities associated with Poland syndrome could potentially either be the inciting cause for the patient’s symptoms or possible predisposing factor for the aforementioned symptoms. Nerve-related symptoms, including weakness, numbness, and tingling, can occur due to the abnormal development of the chest and shoulder muscles, resulting in nerve compression [11,12]. However, further investigation and diagnostic pursuits would be necessary during the course of the hospital admission.

## Conclusions

This case presents Poland syndrome with dextrocardia, a rare congenital symptom, in a patient who presented with a transient ischemic attack. Poland syndrome is not a known cause of stroke, and stroke in the case of a patient with Poland syndrome should be managed according to the general guidelines for the adult population. Although not a known cause of stroke, patients with Poland syndrome should be monitored for the typical risk factors for stroke, such as elevated blood pressure, diabetes, smoking, and elevated cholesterol. A multidisciplinary approach to management of the risk factors should be taken into consideration.
